# Obesity management and continuing medical education in primary care: results of a Swiss survey

**DOI:** 10.1186/1471-2296-12-140

**Published:** 2011-12-22

**Authors:** Carola A Huber, Meichun Mohler-Kuo, Ueli Zellweger, Marco Zoller, Thomas Rosemann, Oliver Senn

**Affiliations:** 1 Institute of General Practice and Health Services Research, University of Zurich, Switzerland; 2 Institute for Social and Preventive Medicine, University of Zurich, Switzerland

## Abstract

**Background:**

The worldwide increase in obesity is becoming a major health concern. General practitioners (GPs) play a central role in managing obesity. We aimed to examine Swiss GPs self-reported practice in diagnosis and treatment of obesity with a special focus on the performance of waist measurement.

**Methods:**

A structured self-reported questionnaire was mailed to 323 GPs recruited from four urban physician networks in Switzerland. Measures included professional experience, type of practice, obesity-related continuing medical education (CME) and practice in dealing with obesity such as waist measurement. We assessed the association between the performance of waist measurement and obesity-related CME by multivariate ordered logistic regression controlling for GP characteristics as potential confounders.

**Results:**

A total of 187 GPs responded to the questionnaire. More than half of the GPs felt confident in managing obesity. The majority of the GPs (73%) spent less than 4 days in the last 5 years on obesity-related CME. More than half of GPs gave advice to reduce energy intakes (64%), intakes of high caloric and alcoholic drinks (56%) and to increase the physical activity (78%). Half of the GPs seldom performed waist measurement and documentation. The frequency of obesity-related CME was independently associated with the performance of waist measurement when controlled for GPs' characteristics by multivariate ordered logistic regression.

**Conclusions:**

The majority of GPs followed guideline recommendations promoting physical activity and dietary counselling. We observed a gap between the increasing evidence for waist circumference assessment as an important measure in obesity management and actual clinical practice. Our data indicated that specific obesity-related CME might help to reduce this gap.

## Background

Obesity is a major health problem associated with increasing risk of diabetes mellitus, hypertension, heart disease, cancer, decreased life expectancy [1] and substantial impact on health-care costs [2,3]. Prevalence of obesity has significantly increased in developed countries over the past two decades [4]. Currently, a national survey in U.S. revealed that more than 65% of adult Americans are classified as either overweight or obese (body mass index [BMI] between 25 to ≥ 35) [5]. The results from recent Swiss Health Survey also showed that overweight and obesity increased considerable in the last 15 years. The overweight and obesity among adult population increased from 30% in 1992 to 37% in 2007 and the increase is mainly due to the increase of overweight [6].

Weight problems were generally determined by body mass index (BMI), but waist circumference could also be a very useful and important indicator to identify those who are at risk and should seek weight management [7,8]. Compared to waist measurement, BMI measurement is not able to differentiate between muscle and fat induced weight increase [9]. Furthermore, it has been shown that the waist circumference is a better predictor of obesity-related health risks such as the risk of metabolic syndrome, hypertension and dyslipidemia [1,6,7,9] than BMI.

The high prevalence of overweight and obesity results in an urgent need for improved obesity-related assessment, treatment and management. General practitioners (GPs) have an important role in preventing and diagnosing weight problems [10,11]. Obese persons were more likely to visit their GP than individuals without obesity [12]. Furthermore, most of patients considered that GPs have a significant role in weight management, have the necessary knowledge and skills to manage weight and consequently would ask their GP for weight loss advice [13]. GPs' practices and attitudes in the management of obesity have been studied in different countries across Europe, but not so far in Switzerland.

The purpose of the present study was to examine GPs' practice in diagnosis and treatment of obesity with a special focus on obesity-related continuous medical education (CME) on the performance of waist measurement. CME is a widely used form of a postgradual learning event assuming a relationship between improved physician knowledge and clinical performance. Furthermore in many countries CMEs provide credits and are part of quality assurance programs for physicians to continue clinical practice.

## Methods

### Study design

The present study is part of an intervention project titled "Management of Obesity and Cardiovascular Risk Factors in Urban Swiss General Practitioners Networks" which aimed to improve GPs' approach in diagnosis and treatment of obesity by a multifaceted intervention program. The program included a baseline assessment, followed by a one-year intervention and a follow-up assessment after the intervention. The intervention was offered to the members of one urban GP network. The members of three other urban networks served as controls. However, the present study reported only the results from the baseline assessment from all four networks focusing on obesity management. All GPs from four urban networks in the German speaking part of Switzerland (168 from the intervention group and 155 from the control group) were eligible to participate.

### Questionnaire and procedure

A self-administered structured questionnaire including 73 questions was developed to assess GPs attitudes, practice, and knowledge as well as management in obesity and cardiovascular risk factors. The items in the questionnaire related to management and treatment are derived from guideline recommendations on obesity [14]. The original questionnaire was modified from a commonly used Australian questionnaire, which was also used among Israeli, French and United States physicians in an adapted form [15-19]. The key independent variable "GPs' obesity related clinical education (CME)" was used as a measure of knowledge and assessed by asking the following question: "How many days did you attend in obesity related CME in the past five years (including literature studies)?" CME was measured on four categories with " < 1 day", "1-3 days", "4-10 days" and " > 10 days". Out of the 73 questions, 10 questions were related to practice (see table 1) and 18 to management (see table 2). Practice is defined as the practice giving advice (i.e. action of counselling) whereas management implies further clinical exams or laboratory measures. The response categories of items related to the practice (10 questions) and the management of obesity treatment (18 questions) were based on a three point Likert-scale and defined as rare practiced (< 10%), occasional practiced (10-50%) and regular practiced (> 50%). In addition, GP characteristics such as age, gender, years of working experience, workload, number of patients a week, percentage of obese patients, percentage of obese patients getting a specific treatment and type of practice (single/group practice) were assessed. Out of the 73 questions, 44 questions were related to GPs attitudes and cardiovascular risk factor management which were not part of the current study.

**Table 1:** GPs' practice in giving advice of weight management

	Frequency of Performance		
	< 10% N (%)	10-50% N (%)	> 50% N (%)
Giving general advice to reduce energy intake	9 (5.1)	55 (30.9)	114 (64.0)
Giving specific information to reduce lipid intake	28 (15.6)	55 (30.6)	97 (53.9)
Giving specific information about carbohydrate and proteins	45 (25.3)	70 (39.3)	63 (35.4)
Individual consultation to reduce the consumption of alcoholic and high caloric drinks	22 (12.4)	56 (31.5)	100 (56.2)
General advice to increase physical activity in everyday life (e.g. walking instead of driving by car)	3 (1.7)	36 (20.1)	140 (78.2)
Advice to do exercises 2 to 3 times a week (e.g. jogging, swimming)	9 (5.0)	52 (29.1)	118 (65.9)
Practical instructions for buying food	106 (58.9)	53 (29.4)	21 (11.7)
Practical instructions for cooking	123 (68.0)	44 (24.3)	14 (7.7)
Urging the patient to use a food diary for 1 week	99 (54.7)	46 (25.4)	36 (19.9)
Clarifying interest and willingness to improve the health status by (support) groups	114 (64.4)	48 (27.1)	15 (8.5)

Percentage may not sum to 100% because of rounding

**Table 2:** GPs' approaches to management and treatment of obesity

	Frequency of Performance		
	< 10% N (%)	10-50% N (%)	> 50% N (%)
Excluding secondary forms of obesity	53 (29.1)	55 (30.2)	74 (40.7)
Annual updating of specific anamnesis and documentation of weight, diets, eating habits and physical activity	50 (27.6)	81 (44.8)	50 (27.6)
Consultations together with the spouse or partner	120 (65.6)	57 (31.2)	6 (3.3)
Asking for weight and physical activity of the children	116 (65.2)	46 (25.8)	16 (9.0)
Assessing and treating eating disorders (e.g. bulimia, binge-eating)	35 (19.2)	83 (45.6)	64 (35.2)
Referring the patient to a psychologist or a psychiatrist in case of mental health problems	56 (31.3)	78 (43.6)	45 (25.1)
Waist measurement and documentation	91 (50.0)	54 (29.7)	37 (20.3)
Total cholesterol measurement	5 (2.8)	31 (17.1)	145 (80.1)
HDL and triglyceride measurement	9 (5.0)	31 (17.1)	141 (77.9)
Assessing the basal metabolic rate and total energy to provide a basis for consultation	128 (71.9)	37 (20.8)	13 (7.3)
Making a total-risk-assessment and discussing the related factors with patients in detail	42 (23.3)	59 (32.8)	79 (43.9)
Applying a valid prognostic tool for this assessment	106 (59.2)	32 (17.9)	41 (22.9)
Systematic evaluation of the patients' motivation and consult the patients about measures	18 (10.0)	83 (46.1)	79 (43.9)
Assessing cognitive skills and education level of the patient	28 (15.7)	77 (43.3)	73 (41.0)
Declaring a common goal and time frame with the patient	13 (7.4)	78 (44.3)	85 (48.3)
Keeping involved in the treatment process if the patient was referred to a specialist	39 (22.3)	91 (52.0)	45 (25.7)
Checking and discussing the achievement of the patient in short intervals (3 to 6 weeks)	12 (6.7)	75 (41.9)	92 (51.4)
Following the treatment improvement over several years	39 (21.7)	93 (51.7)	48 (26.7)

Percentage may not sum to 100% because of rounding

The baseline questionnaires were sent to 323 GPs in March 2006: First, an invitation letter with the questionnaire was sent; second, a reminder postcard was sent two weeks later; finally, a second copy of the questionnaire and a reminding letter was sent to the non-responders another two weeks later.

### Statistical analysis

Descriptive analysis was performed to describe characteristics of GPs as well as their practice and treatment approaches for obesity. Categorical and continuous variables are presented as frequencies and means (SD). The variable of primary interest was the self-reported frequency of GP's performance of waist measurement consisting of three ordered categories (performance in less than 10%, between 10 and 50%, and in more than 50% of obese patients). We assessed the crude association between waist measurement performance and obesity-related CME by using univariate ordered logistic regression.

The odds ratios can be interpreted as a comparison of the chances of the outcome being equal or higher than a specific category as a ratio of the chances of being lower [20].

To further investigate the association between waist measurement performance and obesity-related CME we controlled for GP characteristics as potential confounders by applying multivariate ordered logistic regression modelling. The final model included the following variables, irrespective of a cut-off score for a p-value related to an univariate analysis: sex, age (categorized as younger GPs " < 55 years" vs. older GPs "≥ 55 years"), professional experience (in years), work load (full-time vs. part-time), work setting (working alone vs. working in group), number of patients a week (categorized as " < 100", "100-150", " > 150"), feeling more confident in handling cardiovascular risk factors/obesity due to the membership in networks (categorized as "no", "yes a little", "yes"), estimated proportion of obese patients in the practice (percentage of patients with a BMI > 30 kg/m^2^) and number of days attending obesity related CME in the last five years (categorized as " < 1 day", "1-3 days" and " > 3 days"). The validity of the final model was tested by applying the proportional odds test (Brant test of parallel regression assumption) [21].

Statistical analysis was performed with STATA 10.0 (stata corp.).

## Results

A total of 187 GPs responded to the questionnaire (response rate: 57.8%). The characteristics of the participating GPs are shown in Table 3. The respondents included 144 male (78.3%) and 40 female (21.7%). About half of the GPs (47%) were 55 years old or above and two thirds of them worked full-time (64.3%). On average, GPs reported about 16% (± 10.5) of their patients having an obesity problem (BMI > 30 kg/m^2^) and about 30% (± 26.5) of these obese patients required special treatment according to the assumption of the GPs. More than half of the GPs felt confident in managing cardiovascular disease and/or obesity due to their membership in a physician network. In the past 5 years, most of the GPs (about 74%) attended less than four days of obesity-related CME.

**Table 3:** Characteristics of study participants

	General practitioners (N = 187)	
	N (%)	Mean (standard deviation)
Sex		
Male	144 (78.3)	
Female	40 (21.7)	
Age (in groups)		
< 35 years	1 (0.5)	
35-44 years	28 (15.1)	
45-54 years	69 (37.3)	
≥ 55 years	87 (47.0)	
Professional experience (in years)		17.0 (7.9)
Work load		
Full-time	119 (64.3)	
Part-time 50-90%	56 (30.3)	
Part-time 10-50%	10 (5.4)	
Work setting		
Working alone	49 (35.8)	
Working in group	81 (59.1)	
HMO	4 (2.9)	
Other	3 (2.2)	
No. of patients (a week)		
< 100	101 (54.9)	
100-150	73 (39.7)	
> 150	10 (5.4)	
Percentage of obese patients (BMI > 30 kg/m^2^)		16.2 (10.5)
Percentage of obese patients getting a specific treatment		29.7 (26.5)
Feel more confident in handling cardiovascular risk factors/obesity due to the membership in networks		
Yes	19 (10.4)	
Yes, a little	78 (42.6)	
No	86 (47.0)	
Nr. days attending obesity related CME (past 5 years)		
< 1 day	42 (22.7)	
1-3 days	94 (50.8)	
4-10 days	37 (20.0)	
> 10 days	12 (6.5)	

Percentage may not sum to 100% because of rounding

### Giving advice of weight management and obesity management

Table 1 shows GPs' self reported practice about giving advice of weight management to their patients. More than half of the GPs regularly (> 50%) give advice to increase daily physical activity (78.2%) and 65.9% reported to motivate patients to perform sports. In contrast, most of the GPs rarely (< 10%) gave advise in practical instructions for buying food (58.9%) and cooking (68.0%) and urged the patient to keep a food diary (54.7%).

Table 2 shows GPs' approaches to obesity management. Many of the GPs regularly handled obesity (> 50%) in excluding secondary forms of obesity (40.7%), in total cholesterol measuring (80.1%), in HDL and triglyceride measuring (77.9%) and in checking and discussing the achievement of the patient in short intervals (51.4%). In contrast, many GPs reported rarely (< 10%) asking for weight and physical activity of their children (65.2%), performing waist measurement and documentation (50.0%), assessing the basal metabolic rate and total energy to provide a basis for consultation (71.9%) and applying a valid prognostic tool for this assessment (59.2%).

### Ordered logistic regression analysis

Results of the regression analysis are displayed in Table 4. There was a positive and significant univariate association between the number of days of attending obesity-related CME and the GPs' performance of waist measurement. GPs who attended more than three days of obesity related CME in the past five years were more likely to perform waist measurements (OR: 4.36, p = 0.001) compared to those who attended less than one day of the obesity related CME. This association remained significant when additionally controlled for GP characteristics. The Cragg-Uhler (Nagelkerke) R^2 ^of the final model, a measure of the predictive efficiency, was 8.7%. Checking the final model did not provide evidence that the parallel regression assumption has been violated.

**Table 4:** Ordered logistic regression assessing the crude-and multivariate association between waist measurement^a ^and CME

	OR	95%-CI	p-value
**Crude association**			
Nr. days attending obesity related CME			
< 1 day	1.00		
1-3 days	2.10	0.96-4.54	0.062
> 3 days	4.36	1.85-10.28	0.001
			
Model 1*			
Nr. days attending obesity related CME			
< 1 day	1.00		
1-3 days	2.20	0.81-5.94	0.12
> 3 days	4.18	1.20-12.46	0.014
			
Model 2**			
Nr. days attending obesity related CME			
< 1 day	1.00		
1-3 days	2.14	0.78-5.87	0.14
> 3 days	3.87	1.20-12.46	0.023

^a ^Performing waist measurement: performing 10-50% or > 50% vs. < 10%

*Model 1 adjusted for GP characteristics including age, sex, work load, work setting, professional experience, number of patients a week, feeling more confident in handling cardiovascular risk factors/obesity due to the membership in networks

**Model 2 additionally controlled for percentage of obese patients

## Discussion

The present study examined GPs' characteristics and self-reported practice in obesity management based on a cross-sectional study of 187 GPs in Switzerland. Slightly more than half of the GPs reported that they felt confident in managing obesity. These results are consistent with previous studies which have also found that primary care physicians have confidence in dealing with health consequences of obesity and overweight [16,22].

Recent literature showed that the waist circumference is an important determinant of the cardiovascular risk [6,9]. Due to its easy assessment, it is recommended to measure it in daily practice and document it in the patient file. Although waist measurement has proved to be an efficient measure to predict cardiovascular risk status, our results showed that this procedure is performed only in a minority of cases. Interestingly, significant determinants of performing this procedure are number of days attending obesity related CME. This is in line with Bocquier and colleagues [11] who found that GPs attending CME programmes felt more effective in management of obesity problems, suggesting that CME can help improve GPs' knowledge about treatment and handling of obesity or increase awareness for appropriate risk assessment and handling of patients at risk. Unfortunately we do not have information about what kind of CMEs they attended. Previous studies showed that CME activities are efficient regarding changing behaviour if GPs have to participate actively and have the opportunity to practice skills [23,24]. Our final model revealed a goodness-of-fit of 8.7% (i.e. Cragg-Uhler test) indicating that many other factors affected physician guideline adherence, including external barriers such as patient preferences and environmental factors (i.e. lack of time, resources and reimbursement) that are difficult to overcome [25]. On the other hand the majority of GPs reported to attend obesity related CME for less than four days during the past five years, indicating a potential role of obesity related CME to improve the management in a prevalent condition in primary care patients. However, as many factors affect physician guideline adherence, it is likely that besides CME additional interventions are needed to change behaviour and maintain the changes over time. Combinations of different type of strategies (i.e. multifaceted interventions) may be more effective as they could address a larger variety of barriers for change.

Our study is the first study in Switzerland to evaluate the specific behavioural strategies that GPs advised their obese patients for weight control. Consistent with previous studies [19] and guidelines and recommendation for weight loss [26-28], our results showed that most of the GPs gave advice on lifestyle, dietary and physical activity. Among the recommendations, the most frequently given advice was to increase physical activity. Physical activity was also the most recommended advice in physician surveys in the United States and Israel [17,18,29].

Moreover, our study revealed that most of the GPs recommended various dietary strategies such as reducing energy intake, lipid intake, consumption of alcoholic and high caloric drinks, and carbohydrate and proteins which has also been observed in an Israeli survey where 81% of the family physicians gave always or often advice to decrease total daily calories [17]. Only a minority reported to give detailed instructions for cooking and for buying food as recommended in several guidelines [10,30]. It has to be acknowledged that nutritionists do only marginally exist in Switzerland, so if the GP does not give theses advices, no one will do it.

The main limitation of our results should be acknowledged. Our data reflect self reported behaviours, which can differ from daily practice. Regarding the main result, that CMEs have substantial influence on the reported behaviour, details about attended CMEs were not asked for. Furthermore, we do not have data on non-respondents. However, the participation rate of 57.8% exceeded the participation rate that can be expected from general practice postal surveys thus limiting the risk of a selection bias [31].

One of the strengths of our survey is that the survey included a large number of GPs who are representative to the general Swiss GP population with regard to age, gender and work load according to the annual statistics of the Swiss Medical Association. A main characteristic of our study population is the membership in medical networks. The number of networks, especially in urban areas of Switzerland, steadily increased during recent years reaching up to 48.1% in the year 2010, thus making our study sample comparable to the increasing proportion of GPs joining a network in Switzerland. Furthermore, we confirmed that obesity is a prevalent problem in primary care and obesity related CME has the potential to improve GPs behaviour in obesity management thus our study provides further evidence of the important role of GPs to control for the "obesity epidemic". However, randomised studies that focus on prespecified obesity related CME interventions together with patient related clinical outcomes are needed to further optimize obesity management in clinical practice.

## Conclusions

The majority of GPs followed guideline recommendations promoting physical activity and dietary counselling. We observed a gap between the increasing evidence for waist circumference assessment as an important measure in obesity management and actual clinical practice. Most GPs followed less than 4 days of obesity-related CME during the last 5 years, while 4 or more days of CME did positively affect guideline adherence. Although there remain many factors affecting physician guideline adherence our data indicated that specific obesity-related CME might help to reduce this gap.

## Competing interests

The authors declare that they have no competing interests.

## Authors' contributions

MZ, TR, MMK and UZ contributed to the design of the study. CAH and OS carried out the statistical analysis. CAH and MMK prepared and edited the manuscript. All authors critically reviewed it and contributed to the final manuscript. All authors have seen and approved the final version of the manuscript.

## Pre-publication history

The pre-publication history for this paper can be accessed here:

http://www.biomedcentral.com/1471-2296/12/140/prepub
